# Progress Toward Elimination of Mother-to-Child Transmission of Hepatitis B Virus — Region of the Americas, 2012–2022

**DOI:** 10.15585/mmwr.mm7329a3

**Published:** 2024-07-25

**Authors:** Mary M. Alleman, Leandro Soares Sereno, Alvaro Whittembury, Xi Li, Marcela Contreras, Carmelita Pacis-Tirso, Martha Velandia Gonzalez, Karen Broome, Sandra Jones, Daniel Salas, Monica Alonso, Rania A. Tohme, Annemarie Wasley

**Affiliations:** ^1^Global Immunization Division, Global Health Center, CDC; ^2^Communicable Disease Prevention, Control and Elimination Department, Pan American Health Organization/WHO Regional Office for the Americas, Washington, DC; ^3^The Special Program Comprehensive Immunization, Pan American Health Organization/WHO Regional Office for the Americas, Washington, DC; ^4^The Special Program Comprehensive Immunization, Subregional Program Coordination, Caribbean, Pan American Health Organization/WHO Regional Office for the Americas, Washington, DC; ^5^Communicable Disease Prevention, Control and Elimination Department, Subregional Program Coordination, Caribbean, Pan American Health Organization/WHO Regional Office for the Americas, Washington, DC; ^6^Division of Viral Hepatitis, National Center for HIV, Viral Hepatitis, STD, and TB Prevention, CDC.

**Summary**

**What is already known about this topic?**

In 2022, 5 million persons in the World Health Organization Region of the Americas (AMR) had chronic hepatitis B virus (HBV) infection, the leading cause of hepatocellular carcinoma and cirrhosis. Hepatitis B birth dose (HepB-BD) vaccination followed by 2–3 additional doses (HepB3) during infancy can prevent chronic infection.

**What is added by this report?**

All 51 AMR countries provide HepB3; 67% also provide HepB-BD. Mathematical models suggest that hepatitis B prevalence among children has met the global and regional impact target of ≤0.1% in 14 countries and regionally. HepB3 coverage decreased by ≥10 percentage points in 2022 compared with 2012 in 15 countries; 17 countries do not yet provide HepB-BD.

**What are the implications for public health practice?**

Declines in HepB3 coverage and the absence of HepB-BD in 17 countries’ routine immunization schedules threaten the elimination of mother-to-child transmission of HBV infection throughout AMR. Efforts to introduce HepB-BD and maintain high HepB3 and HepB-BD coverage are needed.

## Abstract

In 2022, an estimated 5 million persons in the World Health Organization Region of the Americas (AMR) were living with chronic hepatitis B virus (HBV) infection, the leading cause of hepatocellular carcinoma and cirrhosis worldwide. Most chronic infections are acquired through mother-to-child transmission (MTCT) or horizontal transmission during childhood and are preventable with hepatitis B vaccination, including a birth dose (HepB-BD), followed by 2–3 additional doses (HepB3) in infancy. The Pan American Health Organization (PAHO) Elimination of MTCT of HBV infection strategy is intended to reduce chronic HBV infection (measured by hepatitis B surface antigen [HBsAg] seroprevalence) to ≤0.1% among children by achieving 1) ≥95% coverage with HepB-BD and HepB3; and 2) ≥80% of pregnant women received testing for HBsAg, and provision of hepatitis B immunoglobulin to HBV-exposed neonates. By 2012, all 51 AMR countries and territories (countries) provided HepB3 nationwide, and by 2021, 34 (67%) provided HepB-BD nationwide. Mathematical models estimate that HBsAg seroprevalence in children is ≤0.1% in 14 (28%) of 51 countries and at the regional level. Three (6%) of 51 countries met the 95% coverage targets for both HepB3 and HepB-BD during both 2021 and 2022. Of these, two have likely met criteria for the elimination of MTCT of HBV infection. However, in 2022, HepB3 coverage had declined by ≥10 percentage points in 15 (37%) of 41 countries with 2012 coverage data for comparison. These declines in HepB3 coverage, as well as the absence of HepB-BD in the routine immunization schedules in 17 countries, threaten PAHO’s progress toward the elimination of MTCT of HBV infection. Efforts to introduce HepB-BD and maintain high HepB3 and HepB-BD coverage are needed.

## Introduction

Globally, chronic hepatitis B virus (HBV) infection is the leading cause of hepatocellular carcinoma and cirrhosis (*1*). In 2022, an estimated 5 million persons in the World Health Organization (WHO) Region of the Americas (AMR)* had chronic HBV infection, and approximately 20,000 died from hepatitis B–related causes (*2*). Most chronic HBV infections are acquired through mother-to-child transmission (MTCT) or horizontal transmission during early childhood (*1*). Infections acquired at age ≤5 years are more likely to become chronic than are those acquired later in life (*1*). To prevent chronic HBV infection, WHO recommends that all infants receive a timely birth dose of hepatitis B vaccine (HepB-BD), defined as receipt within the first 24 hours of life, with 2–3 additional doses (HepB3) preferably administered during the first months of life, simultaneous with vaccines containing diphtheria, tetanus, and pertussis (*1*).

In 1999, the Pan American Health Organization (PAHO) recommended that the 51 countries and territories (countries) in AMR provide HepB3 vaccination for all infants nationwide (universal vaccination) and, in 2011, recommended the inclusion of a universal HepB-BD (*3*,*4*). In 2017, PAHO expanded its strategy for achieving the elimination of MTCT of HIV and syphilis to include HBV infection and Chagas disease (EMTCT Plus) (*5*). PAHO’s EMTCT Plus strategy includes the impact target of reducing hepatitis B surface antigen (HBsAg) seroprevalence (a marker for chronic HBV infection) to ≤0.1% among children aged 4–6 years, and several programmatic targets: 1) achieving high coverage (≥95% nationally and >85% in all provinces or areas) with timely HepB-BD and HepB3; and 2) increasing HBsAg testing among pregnant women and provision of hepatitis B immunoglobulin (HBIG) to HBV-exposed neonates to ≥80% (*5*). The WHO global criteria for the elimination of MTCT of HBV infection are similar and include achieving ≤0.1% HBsAg seroprevalence among children aged ≤5 years^†^ and ≥90% coverage with timely HepB-BD and HepB3 for the two most recent, consecutive years (*6*). This report describes progress toward the elimination of MTCT of HBV infection in AMR during 2012–2022 (*3*,*5*,*6*).

## Methods

### Vaccination Activities

Hepatitis immunization schedules, year of hepatitis vaccine introduction nationwide (universal), and WHO/UNICEF National Immunization Coverage estimates or administrative immunization coverage for timely HepB-BD and HepB3 among children aged <1 year were compiled from PAHO, UNICEF, and WHO immunization data portals, unless otherwise indicated (*3*). WHO/UNICEF National Immunization Coverage estimates are based upon annual country reports submitted via the WHO/UNICEF Joint Reporting Form on Vaccination and coverage surveys.

### HBsAg Seroprevalence

WHO recommends population-based, nationally representative HBsAg serosurveys among children aged ≤5 years to monitor progress toward the elimination of MTCT of HBV infection (*6*). Examples of representative serosurveys (national or subnational) in children or cohorts born after introduction and widespread use of hepatitis B vaccine in the AMR were identified through a search of literature published after 2016 and were reviewed (*3*). Mathematical modeling estimates of HBsAg seroprevalence in children published by the Global Burden of Disease Collaborators,^§^ The Global Health Observatory,^¶^ and the Center for Disease Analysis/Polaris Observatory Collaborators** were reviewed and compiled.

### Additional Indicators for EMTCT Plus

Data on the proportion of pregnant women with at least four prenatal care visits and of births at health facilities were compiled from PAHO’s Core Indicator Portal. Data describing the presence of policies for universal testing for HBV in antenatal care and provision of HBIG to HBV-exposed newborns were compiled from published literature and PAHO and country websites describing strategies for hepatitis B control and the elimination of MTCT of HBV infection. This activity was reviewed by CDC, deemed not research, and was conducted consistent with applicable federal law and CDC policy.^††^

## Results

### Vaccination Activities

**HepB3.** By 2012, all 51 AMR countries provided universal HepB3 vaccination (*3*) (Table 1). Regional HepB3 coverage increased steadily during the 1990s and ranged from 88% to 91% during 2005–2016 (Figure) (*3*). Regional coverage declined to 79% in 2019 but rebounded to 83% in 2022.

**TABLE 1:** Year of introduction of hepatitis B vaccine,*^,^^†^ hepatitis B vaccination schedules,^§^^,^^¶^ and annual estimated or official coverage with third dose of hepatitis B vaccine and a timely hepatitis B birth dose**^,††,§§^ among children aged <1 year, by country or territory, subregion, and region — Region of the Americas, World Health Organization, 2012, 2017, and 2019–2022

Subregion, Country/Territory^¶¶^	Year of introduction		HepB vaccination schedule	HepB3 coverage, %						Timely HepB-BD coverage, %					
	HepB3	HepB-BD		2012	2017	2019	2020	2021	2022	2012	2017	2019	2020	2021	2022
North America**	NA	NA	NA	92	78	79	86	88	89	43	37	41	59	60	62
Canada	1993	1983	Varies by province^§^	70	71	84	84	84	83	NR	NR	NR	NR	NR	NR
Mexico	1999	2007	B, 2, 4, and 6 mos	99	58	56	77	80	83	94^§§^	NR	NR	50	50	50
United States	1991	1991	B,1–2, and 6–18 mos^§^	90	91	91	91	92	93	72	63	67	69	72	75
Central America**	NA	NA	NA	95	91	89	83	81	82	43	60	58	57	55	54
Costa Rica	1997	1997	B, 2, and 6 mos	91	97	94	91	87	94	90	87	87	89	71	71
El Salvador	1999	2015	B, 2, 4, and 6 mos	92	92	90	76	78	75	NA	91	91	90	87	86
Guatemala	2005	2010	B, 2, 4, and 6 mos	96	91	85	83	79	79	35	53	48	48	48	48
Honduras	2000	2007	B, 2, 4, and 6 mos	98	90	88	80	77	78	78	78	78	71	72	69
Nicaragua	1999	NA	2, 4, and 6 mos	98	98	98	92	87	92	NA	NA	NA	NA	NA	NA
Panama	1999	2002	B, 2, 4, and 6 mos	85	81	88	74	87	87	87	87	85	86	87	87
Andean Area**	NA	NA	NA	90	84	83	73	75	76	62	65	67	65	62	62
Bolivia	2000	NA	2, 4, and 6 mos	93	84	75	68	70	69	NA	NA	NA	NA	NA	NA
Colombia^††^	1994	2001	B, 2, 4, and 6 mos	92	92	92	88	86	87	85	81	81	88	87	85
Ecuador	1999	2009	B, 2, 4, and 6 mos	88	85	85	70	68	70	16	61	71	62	61	63
Peru	2003	2003	B, 2, 4, and 6 mos	95	89	88	72	82	82	81	80	82	75	77	79
Venezuela	2000	2008	B, 2, 4, and 6 mos	81	66	64	54	56	56	67	56	52	50	37	37
Southern Cone and Brazil**	NA	NA	NA	95	84	76	78	72	79	80	74	73	64	64	78
Argentina	2000	2000	B, 2, 4, and 6 mos	91	86	83	74	81	81	88	80	77	72	77	77
Brazil	1998	1998	B, 2, 4, and 6 mos	96	82	72	77	68	77	90	80	77	63	62	82
Chile	2005	2019	B, 2, 4, and 6 mos	90	93	96	93	95	96	NA	NA	65	99	98	99
Paraguay^††^	2002	2017	B, 2, 4, and 6 mos	91	91	86	79	70	69	NA	52	NR	NR	NR	NR
Uruguay	1999	NA	2, 4, and 6 mos	95	93	94	92	91	94	NA	NA	NA	NA	NA	NA
Latin Caribbean**	NA	NA	NA	46	77	72	71	71	72	47	49	47	44	42	43
Cuba	1990	1992	B, 2, 4, and 6 mos	96	99	99	99	99	99	99	99	99	99	99	99
Dominican Republic	1994	1997	B, 2, 4, and 6 mos	74	81	87	81	83	87	74	82	81	71	66	71
French Guiana	1994	2008	B, 2, and 11 mos^¶^	NR	NR	NR	NR	NR	NR	NR	NR	NR	NR	NR	NR
Guadeloupe	NR	NR	2, 4, and 11 mos^¶^	NR	NR	NR	NR	NR	NR	NR	NR	NR	NR	NR	NR
Haiti	2012	NA	6, 10, and 14 wks	NR	64	51	51	51	51	NA	NA	NA	NA	NA	NA
Martinique	NR	NR	2, 4, and 11 mos^¶^	NR	NR	NR	NR	NR	NR	NR	NR	NR	NR	NR	NR
Puerto Rico	1994	1999	B, 1–2, and 6–18 mos^§^	NA	NA	73	NA	NA	NA	NA	NA	59	NA	NA	NA
The Caribbean**	NA	NA	NA	92	90	93	86	86	90	5	9	22	24	29	30
Anguilla^††^	1997	2019	B, 2, 4, and 6 mos	100^§§^	89	77	86	79	88	NA	NA	100	100	100	100
Antigua and Barbuda	2000	2021	B, 2, 4, and 6 mos	98	95	99	95	92	99	NA	NA	NA	NA	19	19
Aruba^††^	2003	NA	1, 3, and 9 mos	94^§§^	95	94	NR	92	93	NA	NA	NA	NA	NA	NA
Bahamas	2001	NA	2, 4, and 6 mos	96	94	89	83	86	87	NA	NA	NA	NA	NA	NA
Barbados	2001	NA	2, 4, and 6 mos	87	90	90	85	82	86	NA	NA	NA	NA	NA	NA
Belize	1999	2018	B, 2, 4, and 6 mos	98	88	98	79	83	84	NA	NA	70	67	77	86
Bermuda^††^	1997	NA	6, 7, and 12 mos	92^§§^	81	97	89	100	89	NA	NA	NA	NA	NA	NA
Bonaire	2012	NR	2 mos, 14 wks, 5 mos, and 11 mos^¶^	NR	NR	NR	NR	NR	NR	NR	NR	NR	NR	NR	NR
Cayman Islands^††^	1997	1997	B, 6 wks, and 9 mos	94^§§^	87	NR	79	84	90	NR	NR	NR	75	NR	NR
Curaçao^††^	2011	NR	B, 8, 14, and 22 wks	NR	85	98	NR	NR	NR	NR	NR	NR	NR	NR	NR
Dominica	2006	2017	B, 2, 4, and 6 mos	97	91	99	97	92	92	NA	23	97	99	98	99
Grenada	2001	2017	B, 6–8, 16, and 24 wks	97	96	94	72	77	77	NA	78	96	92	90	91
Guyana	2001	2019	B, 2, 4, and 6 mos	97	97	99	99	98	98	NA	NA	35	49	58	57
Jamaica	2003	NA	6, 12, and 24 wks	96	93	96	95	89	98	NA	NA	NA	NA	NA	NA
Montserrat^††^	1999	2017	B, 2, 4, and 6 mos	94^§§^	100	100	NR	NR	98	NA	100	100	NR	80	83
Saba	2012	NR	2, 3, 4, and 11 mos^¶^	100^§§^	NR	NR	NR	NR	NR	NR	NR	NR	NR	NR	NR
Saint Kitts and Nevis	1999	2015	B, 2, 4, and 6 mos	98	98	97	99	96	96	NA	83	84	93	95	96
Saint Lucia	2002	2018	B, 2, 4, and 6 mos	98	80	92	86	80	81	NA	NA	85	86	94	82
Saint Vincent and the Grenadines	2003	2017	B, 2, 4, and 6 mos	96	99	98	98	99	99	NA	30	99	96	93	93
Sint Eustatius	1997	NR	2, 3, 4, and 11 mos^¶^	NR	NR	NR	NR	NR	NR	NR	NR	NR	NR	NR	NR
Sint Maarten^††^	2000	NA	2, 3, and 6 mos	91^§§^	94	NR	NR	NR	NR	NA	NA	NA	NA	NA	NA
Suriname	2005	2005	B, 2, 4, and 6 mos	76	67	77	51	72	77	51	80	79	79	79	79
Trinidad and Tobago	2003	NA	2, 4, and 6 mos	92	89	93	96	94	93	NA	NA	NA	NA	NA	NA
Turks and Caicos Islands^††^	1999	2019	B, 2, 4, and 6 mos	95^§§^	93	93	84	92	99	NA	NA	NR	NR	NR	100
British Virgin Islands^††^	1999	2016	B, 2, 4, and 6 mos	97^§§^	82	88	87	84	100	NA	90	98	94	NR	91
Total for Region of the Americas**	**NA**	**NA**	**NA**	**91**	**82**	**79**	**81**	**80**	**83**	**56**	**54**	**55**	**60**	**60**	**65**

**Abbreviations:** B = at birth; HepB = hepatitis B–containing vaccine; HepB3 = third dose of HepB; HepB-BD = birth dose of HepB; JRF = World Health Organization/UNICEF Joint Reporting Form on Vaccination; NA = not applicable; NR = data not reported; WUENIC = World Health Organization/UNICEF estimates of national immunization coverage.

* Year of introduction refers to the year the country or territory established universal HepB vaccination (HepB3 or HepB-BD) policies (i.e., HepB vaccination is recommended for all children throughout the country or territory according to schedule).

^†^ All years of HepB3 introduction and all years of HepB-BD introduction before 2016 were compiled from https://www.ncbi.nlm.nih.gov/pmc/articles/PMC5392937. Years of HepB-BD introduction during and after 2016 were compiled from https://data.unicef.org/resources/immunization-country-profiles, https://paho-cim.shinyapps.io/immunization-dashboard, https://immunizationdata.who.int/global/wiise-detail-page/introduction-of-hepb-birth-dose?ISO_3_CODE = PRY&YEAR =, or directly from the JRF.

^§^ HepB vaccination schedules were compiled from https://immunizationdata.who.int/. Canada’s provinces have varying schedules; some include a birth dose, and some initiate vaccination after 1 year of life. Details by Canadian province are available online (https://www.canada.ca/en/public-health/services/provincial-territorial-immunization-information.html). For the United States (including Puerto Rico), the HepB vaccination schedule was extracted from https://www.cdc.gov/vaccines/schedules/downloads/child/0-18yrs-child-combined-schedule.pdf.

^¶^ If not available at https://immunizationdata.who.int/ or as indicated in footnote above, HepB vaccination schedules were compiled from https://www.ncbi.nlm.nih.gov/pmc/articles/PMC5392937.

** Annual HepB3 and HepB-BD national coverage values are WUENIC prepared using data from the JRF; they were compiled from https://immunizationdata.who.int. Timely administration of HepB-BD is defined as administration within 24 hours of birth. For the United States, coverage estimates were determined as follows for HepB3 and HepB-BD: 2016–2018 estimates are among children aged 19–35 months during the previous survey year (e.g., 2018 estimate is from survey year 2017 data); 2019–2022 estimates are among children by age 24 months. The 2019 estimate is from combined 2015–2016 birth cohort, the 2020 estimate is from combined 2016–2017 birth cohort, the 2021 estimate is from combined 2017–2018 birth cohort, and the 2022 estimate is from combined 2018–2019 birth cohort. For Puerto Rico, HepB3 estimates are among children by age 24 months, by birth cohort; HepB-BD estimates are among children on the first day of life, by birth cohort. Data were not collected in Puerto Rico during some of the reporting years. In addition, even when data were collected, sample size was sometimes too small to calculate reliable coverage estimates. HepB3 and HepB-BD subregional and regional coverage estimates were generated using data from the 10-year period (2012–2022) for the 51 countries or territories using WUENIC estimates or official coverage. Countries or territories not reporting coverage or that have not adopted universal HepB-BD vaccination policies were assumed to have no coverage. HepB3 and HepB-BD subregional and regional coverage values were weighted coverage based upon the number of average annual births for 2012–2022, as available, from the United Nations population estimates.

^††^ Where annual WUENIC were not available, values are from official or administrative national immunization coverage as reported on the country or territory’s JRF (https://paho-cim.shinyapps.io/immunization-dashboard). Any reported coverage exceeding 100% was considered 100%.

^§§^ Where annual WUENIC and official or administrative national immunization coverage as reported on the country or territory’s JRF were not available, values are those provided in a previous report. https://www.ncbi.nlm.nih.gov/pmc/articles/PMC5392937

^¶¶^ Vaccination data for some territories within the Region of the Americas are not consistently reported to the World Health Organization separately from their associated countries.

**FIGURE:**
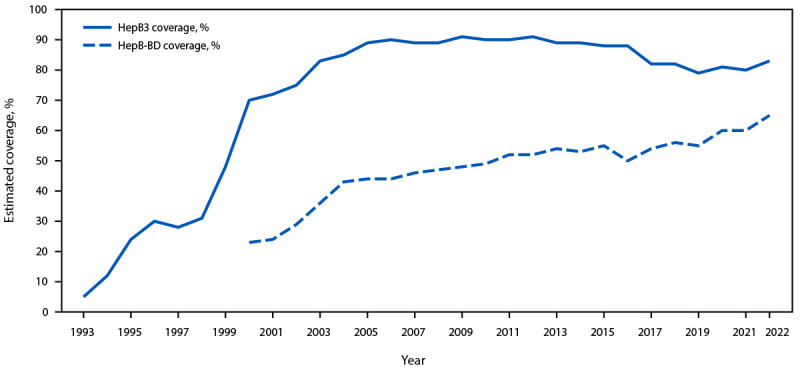
Annual estimated coverage with the third dose of hepatitis B vaccine and timely hepatitis B birth dose* among children aged <1 year — Region of the Americas, World Health Organization, 1993–2022 **Abbreviations:** HepB3 = third dose of hepatitis B–containing vaccine; HepB-BD = birth dose of hepatitis B monovalent vaccine; WHO = World Health Organization. * Regional coverage values are based upon WHO/UNICEF National Immunization Coverage estimates and were compiled from the WHO immunization data portal. https://immunizationdata.who.int

HepB3 coverage in 2022 declined by ≥13 percentage points from that in 2012 in the Andean Area, Central America, and Southern Cone and Brazil subregions (Table 1) and declined by ≥10 percentage points in 15 (37%) of 41 countries reporting data for both 2012 and 2022 (*3*). Coverage in Haiti and Suriname never exceeded 68% and 81%, respectively, during the reporting period. Twelve countries met the global target of ≥90% HepB3 coverage during both 2021 and 2022; among those, five met the PAHO target of ≥95% coverage.

**HepB-BD.** As of 2021, 34 (67%) countries provided universal HepB-BD vaccination (Table 1) (*3*). During 2000–2022, regional HepB-BD coverage increased from 23% to 65% (Figure) (*3*) and during 2012–2022, remained relatively stable or increased in all subregions. Among 15 countries reporting HepB-BD coverage for 2012 and 2022, coverage declined in 2022 by ≥10 percentage points in Argentina, Costa Rica, Mexico, and Venezuela compared with that in 2012 (*3*). Seven countries met the global target of ≥90% HepB-BD coverage during both 2021 and 2022; among those countries, five met the PAHO target of ≥95% coverage.

### HBsAg Seroprevalence

Estimates from three mathematical models suggest that regional HBsAg seroprevalence among children aged ≤5 years is <0.1% (Table 2). Among 26 countries for which three modeled estimates are available, the estimated seroprevalence from all three models is ≤0.1% in 14 (54%) countries, among which two (Chile and Cuba) reported both HepB3 and HepB-BD coverage ≥95% during both 2021 and 2022. Recently published nationally representative HBsAg serosurveys that included children and vaccine-eligible cohorts conducted in Haiti, Mexico, and the United States corroborate the 2022 estimates for these countries. In addition, recently published HBsAg serosurvey results from population-based subnational surveys conducted in AMR show that decades of vaccination against HBV have led to reductions in the seroprevalence of HBsAg among cohorts that have been age-eligible for vaccination compared with seroprevalence among older cohorts.

**TABLE 2:** Estimated hepatitis B surface antigen seroprevalence among children aged <5 years, coverage of reproductive health and maternal and child health services, and policies of interventions for the prevention of mother-to-child transmission of hepatitis B, by country or territory and region — Region of the Americas, World Health Organization, 2008–2022

Subregion, Country/Territory	Estimated HBsAg seroprevalence among children aged ≤5 years, % (95% CI)*			Estimated antenatal care coverage with ≥4 visits, % (yr of data)**	Estimated births at health facilities, % (yr of data)**	Policy	
	Global Burden of Disease collaborators,^†^ 2019	The Global Health Observatory,^§^ 2020	Center for Disease Analysis/Polaris Observatory collaborators,^¶^ 2022			Universal HBV testing in antenatal care, 2020–2022^††^	Provision of HBIG to HBV-exposed newborns, 2020^§§^
**North America**							
Canada^¶¶^	0.3 (0.2–0.3)	0.34 (0.29–0.39)	<0.1 (<0.1–<0.1)	99.0 (2020)	NR	Yes	Yes
Mexico***	0.02 (0.01–0.02)	0.03 (0.02–0.04)	<0.1 (<0.1–<0.1)	89.6 (2022)	NR	NR	Yes
United States***	0.03 (0.02–0.04)	0.01 (0.01–0.02)	<0.1 (<0.1–<0.1)	95.4 (2021)	97.8 (2021)	Yes	Yes
**Central America**							
Costa Rica	0.02 (0.02–0.03)	0.02 (0.01–0.03)	<0.1 (<0.1–<0.1)	94.1 (2022)	98.9 (2022)	Yes	Yes
El Salvador	0.07 (0.05–0.10)	0.02 (0.01–0.07)	<0.1 (<0.1–<0.1)	87.2 (2021)	99.7 (2022)	No	No
Guatemala	0.2 (0.1–0.2)	0.03 (0.02–0.04)	<0.1 (<0.1–<0.1)	43.0 (2014)	71.0 (2021)	Yes	No
Honduras	0.1 (0.1–0.2)	0.03 (0.01–0.11)	<0.1 (<0.1–<0.1)	89.0 (2012)	61.4 (2022)	No	No
Nicaragua	0.03 (0.03–0.03)	0.09 (0.05–0.17)	<0.1 (<0.1–0.1)	91.9 (2022)	96.3 (2022)	NR	NR
Panama	0.08 (0.05–0.11)	0.07 (0.05–0.09)	<0.1 (<0.1–<0.1)	88.2 (2019)	91.5 (2021)	Yes	No
**Andean Area**							
Bolivia	0.03 (0.02–0.04)	0.14 (0.05–0.29)	<0.1 (<0.1–<0.1)	81.3 (2022)	93.1 (2022)	NR	NR
Colombia^¶¶^	0.3 (0.2–0.3)	0.15 (0.12–0.19)	<0.1 (<0.1–<0.1)	81.6 (2021)	97.3 (2021)	Yes	Yes
Ecuador	0.04 (0.03–0.06)	0.09 (0.03–0.33)	<0.1 (<0.1–0.1)	79.0 (2013)	96.0 (2020)	Yes	Yes
Peru^¶¶^	0.04 (0.03–0.05)	0.06 (0.05–0.07)	<0.1 (<0.1–<0.1)	85.1 (2022)	93.3 (2022)	Yes	Yes
Venezuela	0.1 (0.1–0.2)	0.15 [0.12–0.18]	0.2 (0.2–0.4)	82.6 (2018)	NR	No	Yes
**Southern Cone and Brazil**							
Argentina	0.02 (0.01–0.03)	0.01 (0.01–0.02)	<0.1 (<0.1–<0.1)	72.8 (2021)	97.8 (2021)	Yes	Yes
Brazil	0.1 (0.1–0.2)	0.03 (0.02–0.03)	<0.1 (<0.1–<0.1)	92.9 (2022)	98.9 (2021)	Yes	Yes
Chile	0.02 (0.01–0.03)	0.03 (0.02–0.05)	<0.1 (<0.1–<0.1)	NR	99.6 (2021)	Yes	Yes
Paraguay	0.1 (0.1–0.2)	0.42 (0.09–2.08)	<0.1 (<0.1–<0.1)	79.6 (2021)	NR	Yes	Yes
Uruguay	0.01 (0.01–0.02)	0.15 (0.02–1.21)	NA	97.2 (2022)	100.0 (2021)	Yes	Yes
**Latin Caribbean**							
Cuba	0.02 (0.02–0.03)	0.03 (0.01–0.05)	<0.1 (<0.1–<0.1)	79.3 (2019)	99.8 (2022)	Yes	Yes
Dominican Republic	0.2 (0.1–0.2)	0.1 (0.03–0.37)	0.1 (<0.1–0.1)	92.6 (2019)	99.9 (2021)	NR	NR
French Guiana	NA	NA	NA	84.5 (2016)	NR	NR	NR
Guadeloupe	NA	NA	NA	NR	NR	NR	NR
Haiti***	0.3 (0.2–0.4)	1.04 (0.75–1.41)	0.4 (0.3–0.8)	67.0 (2012)	67.3 (2021)	No	No
Martinique	NA	NA	NA	98.5 (2016)	NR	NR	NR
Puerto Rico	0.04 (0.03–0.05)	NA	NA	97.8 (2020)	98.8 (2020)	NR	NR
**The Caribbean**							
Anguilla	NA	NA	NA	100.0 (2012)	100.0 (2022)	Yes	Yes
Antigua and Barbuda	0.03 (0.02–0.04)	0.19 (0.07–0.68)	NA	75.0 (2022)	99.0 (2022)	Yes	Yes
Aruba	NA	NA	NA	100.0 (2020)	NR	NR	NR
Bahamas	0.03 (0.02–0.04)	0.16 (0.01–4.56)	NA	81.2 (2019)	99.2 (2022)	Yes	Yes
Barbados	0.03 (0.02–0.05)	0.18 (0.05–0.55)	NA	90.0 (2020)	98.2 (2022)	Yes	No
Belize	0.07 (0.05–0.09)	0.6 (0.46–0.76)	<0.1 (<0.1–<0.1)	NR	92.1 (2022)	Yes	No
Bermuda	0.2 (0.2–0.3)	NA	NA	98.0 (2021)	99.8 (2021)	Yes	Yes
Bonaire	NA	NA	NA	NR	NR	NR	NR
Cayman Islands	NA	NA	NA	97.0 (2017)	NR	Yes	Yes
Curaçao	NA	NA	NA	NR	NR	NR	NR
Dominica	0.04 (0.02–0.05)	0.2 (0.06–0.6)	NA	95.0 (2020)	99.0 (2022)	Yes	Yes
Grenada	0.06 (0.04–0.08)	0.12 (0.04–0.42)	NA	69.0 (2021)	NR	Yes	Yes
Guyana	0.05 (0.03–0.07)	0.4 (0.07–2.11)	0.1 (<0.1–0.2)	95.0 (2022)	NR	Yes	No
Jamaica	0.01 (0.01–0.02)	0.55 (0.37–0.79)	0.2 (0.1–0.3)	87.0 (2008)	NR	No	Yes
Montserrat	NA	NA	NA	100.0 (2022)	NR	NR	NR
Saba	NA	NA	NA	NR	NR	NR	NR
Saint Kitts and Nevis	0.03 (0.02–0.04)	0.06 (0.02–0.22)	NA	85.0 (2022)	100.0 (2022)	NR	NR
Saint Lucia	0.05 (0.04–0.07)	0.22 (0.06–0.77)	NA	90.0 (2022)	99.0 (2022)	NR	NR
Saint Vincent and the Grenadines	0.02 (0.01–0.03)	0.15 (0.05–0.49)	NA	NR	NR	NR	NR
Sint Eustatius	NA	NA	NA	NR	NR	NR	NR
Sint Maarten	NA	NA	NA	100.0 (2018)	NR	NR	NR
Suriname	0.04 (0.03–0.05)	0.07 (0.02–0.28)	0.1 (0.1–0.2)	66.8 (2010)	NR	Yes	Yes
Trinidad and Tobago	0.06 (0.04–0.08)	0.19 (0.04–0.81)	0.1 (0.1–0.2)	100.0 (2017)	NR	NR	NR
Turks and Caicos Islands	NA	NA	NA	57.7 (2022)	100.0 (2022)	Yes	Yes
British Virgin Islands	0.1 (0.1–0.1)	NA	NA	100.0 (2017)	100.0 (2022)	Yes	NR
**Region of the Americas**	**0.08 (0.06**–**0.11)**	**0.07 (0.05**–**0.13)**	**<0.1 (<0.1**–**<0.1)**	**—**	**—**	**—**	**—**

**Abbreviations:** HBIG = hepatitis B immune globulin; HBsAg = hepatitis B surface antigen; HBV = hepatitis B virus; NA = not available; NR = not reported.

* Data are presented in the format of original publication from referenced sources; thus, formatting within and between columns might differ.

^†^ https://www.thelancet.com/journals/langas/article/PIIS2468-1253(22)00124-8/fulltext

^§^ https://www.who.int/data/gho/data/indicators/indicator-details/GHO/hepatitis-b-surface-antigen-(hbsag)-prevalence-among-children-under-5-years

^¶^ https://cdafound.org/polaris-countries-database/; https://www.sciencedirect.com/science/article/abs/pii/S2468125323001978?via%3Dihub

** https://opendata.paho.org/en/core-indicators/core-indicators-dashboard

^††^ Universal testing for HBV in antenatal care is the HBsAg testing of all women seeking antenatal care for the purposes of determining their eligibility for antiviral treatment during pregnancy and for the provision of HBIG to the exposed newborn (https://www.who.int/publications/i/item/9789240090903 [Accessed May 9, 2024]). Data are from Towards the Elimination of HIV, Syphilis, Hepatitis B, and Chagas disease in the Americas, EMTCT Plus Initiative 2010–2021, Annex Tables 1 and 4 (https://www.paho.org/en/documents/fact-sheet-emtct-plus-initiative-2011-2021-towards-elimination-hiv-syphilis-hepatitis-b); https://www.canada.ca/en/public-health/services/publications/healthy-living/canadian-immunization-guide-part-4-active-vaccines/page-7-hepatitis-b-vaccine.html; https://www.sciencedirect.com/science/article/pii/S0749379723000569?via%3Dihub; https://www.cdc.gov/hepatitis/statistics/2022surveillance/perinatal-hepatitis-b.htm [All accessed May 24, 2024].

^§§^ HBIG prophylaxis provided within 24 hours of birth, in conjunction with hepatitis B birth dose vaccination, might be of additional benefit for infants whose mothers are HBsAg-positive, particularly if they have high levels of HBV DNA (https://www.who.int/publications/i/item/9789240090903). Data are from Towards the Elimination of HIV, Syphilis, Hepatitis B, and Chagas disease in the Americas, EMTCT Plus Initiative 2010–2021, Annex Tables 1 and 4 (https://www.paho.org/en/documents/fact-sheet-emtct-plus-initiative-2011-2021-towards-elimination-hiv-syphilis-hepatitis-b) or through direct consultation with countries in 2020.

^¶¶^ Subnational HBsAg serosurveys targeting high-risk areas that enrolled representative samples of children or vaccine-eligible cohorts illustrate the impact of vaccination against hepatitis B are as follows. Canada: https://pubmed.ncbi.nlm.nih.gov/28736196; Colombia: https://www.ncbi.nlm.nih.gov/pmc/articles/PMC10200037; https://www.sciencedirect.com/science/article/pii/S0264410X17315463?via%3Dihub; https://pubmed.ncbi.nlm.nih.gov/28780978/; and Peru: https://www.ncbi.nlm.nih.gov/pmc/articles/PMC7410281/pdf/pone.0236993.pdf; https://rpmesp.ins.gob.pe/index.php/rpmesp/article/view/4696/3681; https://www.medigraphic.com/pdfs/salpubmex/sal-2020/sal203b.pdf

*** Nationally representative HBsAg serosurveys are as follows. Haiti: https://www.ncbi.nlm.nih.gov/pmc/articles/PMC6609174; Mexico: https://www.ncbi.nlm.nih.gov/pmc/articles/PMC6422518/pdf/khvi-15-02-1533617.pdf; and the United States: https://www.ncbi.nlm.nih.gov/pmc/articles/PMC10069827; https://pubmed.ncbi.nlm.nih.gov/26251317

### Additional Indicators for EMTCT Plus

According to reports received by PAHO from 35 countries, as of 2020, 19 (54%) had national goals for the elimination of MTCT of HBV infection (*5*). Forty-three countries reported data on prenatal care visits by pregnant women; in 21 (49%) countries, ≥90% of pregnant women had at least four prenatal visits. In 27 of 30 (90%) countries with data on delivery location, ≥91.5% of births were at health facilities. Twenty-seven (84%) of 32 countries with data reported providing universal antenatal HBV testing, and 24 (75%) of 32 reported providing HBIG for neonates born to mothers with high levels of HBV DNA; however, the extent of coverage with these interventions is unknown in many AMR countries.

## Discussion

Substantial progress has been made toward the elimination of MTCT of HBV infection in AMR. PAHO has supported vaccination against hepatitis B in the region since the 1990s by 1) advocating for vaccination to stakeholders, 2) providing technical support for the development of national vaccination policies, 3) building health care worker capacity, and 4) facilitating vaccine procurement.^§§^ Mathematical models estimate the prevalence of chronic HBV infection among children aged ≤5 years, as measured by HBsAg seroprevalence, to be <0.1% regionally, and 14 countries met both regional and global impact targets for the elimination of MTCT of HBV infection (*5*,*6*). Among the 14 countries identified as likely to have met the HBsAg seroprevalence target, two reported HepB-BD and HepB3 coverage ≥95% during both 2021 and 2022, meeting both the regional and global programmatic targets for the elimination of MTCT of HBV infection, and both implemented antenatal and maternal and child health policies supporting the elimination of MTCT of HBV infection (*5*,*6*).

PAHO has endorsed a process for validating achievement of the elimination of MTCT of HBV infection (*6*), and regional and national validation committees have been established. Because countries are evaluated for the elimination of MTCT of HBV infection, representative seroprevalence data documenting the prevalence of chronic HBV infection in children are needed. Innovative approaches, such as the integration of HBsAg testing into other surveys or sampling focused on geographic areas with documented high risk for HBV infection such as the two-phase method for verifying the elimination of MTCT of HBV infection used in Colombia (*7*), might facilitate the collection of essential data.

Despite regional progress, an estimated 34,000 children aged ≤5 years in the Americas had chronic HBV infection in 2022 (*8*). Few countries are consistently achieving the ≥90% HepB3 global coverage target. Declines in HepB3 coverage during 2012–2022 threaten progress toward elimination of chronic HBV infection in children. These declines have been attributed to inadequate sustainable financing and reductions in social mobilization for vaccination, increasing vaccination hesitancy, insecurity linked to civil unrest, lack of easy access to health services for some populations, and recently, the COVID-19 pandemic and consequent health service disruptions^¶¶^^,^***^,^^†††^^,^^§§§^^,^^¶¶¶^ (*9*,*10*). To overcome these constraints and improve HepB3 vaccination coverage, PAHO is working with countries to implement the recommendations in the 2021 Reinvigorating Immunization as a Public Good for Universal Health resolution**** and the new Regional Immunization Action Plan 2030.^††††^

Although most children born in AMR live in countries with routine HepB-BD, 17 countries, particularly in the Caribbean and Latin Caribbean subregions (13 of the 17), have not introduced universal birth dose vaccination (*3*). In countries with HepB-BD, efforts to address disparities in coverage and access and to ensure timely administration will protect infants at risk for HBV infection (*1*). Most births in the region occur at health facilities; thus, implementation of policies such as standing orders for newborn HepB-BD vaccination before discharge of mother and child, paired with education of pregnant women and maternal and child health care staff members about the importance of the birth dose, can improve timely administration and coverage.

The region continues to expand efforts to achieve the elimination of MTCT of HBV infection by integrating antenatal viral testing, antiviral treatment during pregnancy when indicated, and provision of HBIG for HBV-exposed newborns into the established platforms providing interventions for the elimination of MTCT of HIV and syphilis (*5*). PAHO’s Strategic Fund is tasked with improving access to and reducing costs of hepatitis B–relevant health supplies and medicines for the region.^§§§§^

### Limitations

The findings in this report are subject to at least two limitations. First, current HepB-BD and HepB3 vaccination schedules and coverage or the elimination of MTCT programmatic indicators were not available for all countries or all years, limiting the completeness of summaries on regional progress on the elimination of MTCT of HBV infection. Second, not all countries have systems that differentiate reporting of timely versus any HepB-BD administration, thus potentially overestimating timely birth dose coverage.

### Implications for Public Health Practice

Although progress has been made, declines in HepB3 coverage and the absence of HepB-BD introduction in 17 countries threaten PAHO’s progress toward the elimination of MTCT of HBV infection. To advance toward the regional goal of the elimination of MTCT of HBV infection, continued efforts are needed to support HepB-BD introduction and the achievement and maintenance of high HepB-BD and HepB3 coverage.

## Footnotes

* PAHO/AMR consists of 51 countries and territories (referred to as countries in the text) as follows. Countries (35): Antigua and Barbuda, Argentina, Bahamas, Barbados, Belize, Bolivia, Brazil, Canada, Chile, Colombia, Costa Rica, Cuba, Dominica, Dominican Republic, Ecuador, El Salvador, Grenada, Guatemala, Guyana, Haiti, Honduras, Jamaica, Mexico, Nicaragua, Panama, Paraguay, Peru, St. Lucia, St. Vincent and the Grenadines, St. Kitts and Nevis, Suriname, Trinidad and Tobago, United States, Uruguay, and Venezuela; and territories (16): Anguilla, Aruba, Bermuda, Bonaire, Cayman Islands, Curaçao, French Guiana, Guadeloupe, Martinique, Montserrat, Puerto Rico, Saba, Sint Eustatius, Sint Maarten, Turks and Caicos Islands, and British Virgin Islands.

† Globally, WHO has defined the target as ≤0.1% HBsAg seroprevalence in children aged ≤5 years. In countries with a long history of sustained, high hepatitis B vaccination coverage, flexibility exists to conduct surveys among children aged >5 years. In AMR, PAHO has set the target age group for hepatitis B serosurveys to be children aged 4–6 years.

§ https://www.healthdata.org/research-analysis/gbd (Accessed July 2, 2024).

¶ https://www.who.int/data/gho (Accessed July 2, 2024).

**
https://cdafound.org/polaris/

†† 45 C.F.R. part 46, 21 C.F.R. part 56; 42 U.S.C. Sect. 241(d); 5 U.S.C. Sect. 552a; 44 U.S.C. Sect. 3501 et seq.

§§
https://www.paho.org/en/revolving-fund

¶¶
https://www.paho.org/en/news/20-4-2023-risk-vaccine-preventable-disease-outbreaks-30-year-high-paho-director-says

***
https://www.unicef.org/lac/en/press-releases/1-in-4-children-in-latin-america-and-caribbean-is-missing-out-life-saving-vaccines

†††
https://www.ijidonline.com/article/S1201-9712(19)30143-2/pdf

§§§
https://www.connectas.org/the-silent-backslide-of-childhood-vaccination-in-latin-america/

¶¶¶
https://www.paho.org/en/news/27-7-2021-disruption-health-services-during-covid-19-pandemic-threatens-elimination-hepatitis

****
https://www.paho.org/en/documents/ce16814-reinvigorating-immunization-public-good-universal-health

††††
https://www.paho.org/en/events/webinar-regional-immunization-action-plan-americas-2030

§§§§
https://www.paho.org/en/paho-strategic-fund
